# *Choosing Wisely* Statins

**DOI:** 10.20945/2359-3997000000312

**Published:** 2020-11-27

**Affiliations:** 1 Instituto Estadual de Diabetes e Endocrinologia Rio de Janeiro RJ Brazil Instituto Estadual de Diabetes e Endocrinologia (IEDE), Rio de Janeiro, RJ, Brazil

Cardiovascular diseases (CVDs) are the leading cause of death worldwide ( 1 ). Therefore, CVD prevention should be considered in routine clinical practice for all adults, particularly those with comorbidities that increase the risk of cardiovascular events, including diabetes mellitus (DM), chronic kidney disease (CKD), and hypertension. Statins are among the medications with the highest level of evidence in preventing CVDs, particularly in patients at increased cardiovascular risk and those with a history of CVD *(i.e* ., secondary prevention). Considering all the different statins available in the market (fluvastatin, atorvastatin, rosuvastatin, pravastatin, lovastatin, pitavastatin, simvastatin), how should physicians choose the right statin for each patient? Moreover, is there enough evidence to suggest that certain statins should be prescribed in the presence of specific comorbidities?

*Choosing Wisely* is an initiative by the American Board of Internal Medicine (ABIM) “that seeks to advance a national dialogue on avoiding unnecessary medical tests, treatments and procedures” ( 2 ). In line with the initiative, Borges and cols. published an interesting review in this edition of the *Archives of Endocrinology and Metabolism* with the aim of rationalizing statin treatment to reduce side effects and improve adherence ( 3 ). However, how simple is it to choose statins wisely for individual patients? Should the decision be based on evidence, efficacy, or safety?

Patients with a history of a cardiovascular event ( *i.e* ., secondary prevention) are those who benefit most from statins. In these patients, the decision is quite simple: they should be prescribed a high potency statin, either atorvastatin 40-80 mg or rosuvastatin 20-40 mg, with no major differences between both. In the review, Borges and cols. recommend atorvastatin to be the preferred statin in two situations, namely, CKD and heart failure with preserved ejection fraction (HFpEF). However, is there enough evidence to support this recommendation? For patients with HFpEF, there is no single evidence supporting the use of any statin at all. For patients with CKD, on the other hand, two different systematic reviews have demonstrated that statins may slow the rate of glomerular filtration rate (GFR) changes and decrease proteinuria ( 4 , 5 ). These effects seem to be more pronounced with atorvastatin, although the results derive from a single study. In conclusion, the wise choice for secondary prevention seems to be a high intensity statin, regardless of the presence of comorbidities.

The choice of statin for patients without a history of cardiovascular events ( *i.e* ., primary prevention) is more complex and requires additional individualization. Borges and cols. elegantly discussed this aspect in their review. Several statins can be prescribed for these patients, and the presence of comorbidities can drive the choice. However, patients often have more than one comorbidity. Those at increased risk of DM frequently have nonalcoholic fatty liver disease. Elderly patients usually have low estimated GFR or even CKD. Patients with HIV using antiretroviral therapy have an increased risk of DM and often present nonalcoholic steatohepatitis. How should we proceed in these cases? How can we choose a statin while taking into account so many different comorbidities? Specific answers to these questions are complex, and the only way to rationalize them is by resorting to knowledge. Physicians must understand the differences and similarities between statins to be able to make a wise choice for the best statin for each patient. This is the concept of Personalized Medicine. We must be able to use the best available evidence to find the best intervention for each of our patients. In line with this thought, the review of Borges and cols. is greatly helpful.

Another important example of Personalized Medicine is in an interesting article by Rissetti and cols. in patients with hypopituitarism treated with different simvastatin doses compared with controls ( 6 ). Patients with hypopituitarism often present several comorbidities, including dyslipidemia. Although only simvastatin was used in the study by Rissetti and cols., patients with hypopituitarism exhibited a similar decrease in LDL-cholesterol levels as those in the control group. In case these patients with hypopituitarism have a history of cardiovascular events, shouldn't they be switched to a higher intensity statin? In this specific population, in case of impaired glucose tolerance or age above 65 years, should we consider different statins?

Seven statins are currently available in the market, each with specific characteristics in terms of potency, pharmacokinetics, drug interaction, metabolization, and excretion. Each statin has been analyzed in specific studies and specific populations, and ezetimibe has not even been included in the discussion. Due to the high prevalence of dyslipidemia, physicians should be able to recognize which characteristics of the patient match each statin to decrease the side effects and improve the adherence to these medications. This means to follow the principle of *“primum non nocere et in dubio abstine* ”. Knowledge is the only path to a wise choice.
